# Phase II prefusion non-stabilised Covid-19 mRNA vaccine randomised study

**DOI:** 10.1038/s41598-023-49653-6

**Published:** 2024-01-29

**Authors:** Thanyawee Puthanakit, Eakachai Prompetchara, Sivaporn Gatechompol, Chutitorn Ketloy, Arunee Thitithanyanont, Anan Jongkaewwattana, Supranee Buranapraditkun, Sasiwimol Ubolyam, Stephen J. Kerr, Jiratchaya Sophonphan, Tanakorn Apornpong, Wonngarm Kittanamongkolchai, Sarawut Siwamogsatham, Somchai Sriplienchan, Kanitha Patarakul, Tuangtip Theerawit, Pathariya Promsena, Rapisa Nantanee, Siwaporn Manomaisantiphap, Sarun Chokyakorn, Lina Hong, Mijo Samija, David C. Montefiori, Hongmei Gao, Amanda Eaton, Wassana Wijagkanalan, Mohamad-Gabriel Alameh, Drew Weissman, Kiat Ruxrungtham, ChulaVac001-Phase 2 study team, Monta Tawan, Aungsumalin Sutjarit, Thutsanun Meepuksom, Jitthiwa Athipunjapong, Thidarat Jupimai, Juthamanee Moonwong, Rachaneekorn Nadsasarn, Sasiprapha Khamthi, Pornpavee Nuncharoen, Yanisar Chanpoom, Phattharapa Khamkhen, Nirunya Narupan, Siriwan Thongthip, Konsiri Soisoongnern, Chomnid Shanyip, Thanakan Rachpradit, Kanipha Sriphraram, Wassana Somhanwong, Timporn Boondamnern, Nittaya Boonnak, Nitiya Chomchey, Somporn Tipsuk, Suwanna Puttamaswin, Siriyapat Yewande, Plengsri Lertarom, Anuntaya Uanithirat, Anongnart Anuchadbut, Sararut Chanthaburanun, Katawut Tarawat, Apicha Mahanontharit, Wanida Sinthon, Sasitorn Plakunmonthonw, Suwat Wongmueang, Theera Dalodom, Bunruan Sopa, Nuchthida Phongam, Anchisa Sri-Arunsak, Umaporn Chobkarching, Channuwat Bouko, Sukanya Junseeha, Boonsri Phuphalicho, Palida Pingthaisong, Apichaya Khlaiphuengsin, Patcharee Pararit, Patcharin Eamyoung, Thitiporn Somjit, Thatri Iampornsin, Dutmanee Thongchomphunut, Suwimon Manopwisedjaroen, Thanida Laopanupong, Supanuch Ekronarongchai, Chanya Srisaowakarn, Yuparat Jantraphakorn, Kanjana Srisutthisamphan, Ponsuk Visudhipan Grandin

**Affiliations:** 1 Department of Pediatrics, Faculty of Medicine, Chulalongkorn University, Bangkok, Thailand; 2 Center of Excellent for Pediatric Infectious Diseases and Vaccines, Faculty of Medicine, Chulalongkorn University, Bangkok, Thailand; 3 Maha Chakri Sirindhorn Clinical Research Center (ChulaCRC), Faculty of Medicine, Chulalongkorn University, Bangkok, Thailand; 4 Center of Excellence in Vaccine Research and Development (Chula VRC), Faculty of Medicine, Chulalongkorn University, Bangkok, Thailand; 5 Department of Laboratory Medicine, Faculty of Medicine, Chulalongkorn University, Bangkok, Thailand; 6 School of Global Health, Faculty of Medicine, Chulalongkorn University, Bangkok, Thailand; 7 Center of Excellence in Tuberculosis, Faculty of Medicine, Chulalongkorn University, Bangkok, Thailand; 8 HIV-NAT, Thai Red Cross AIDS Research Centre, Bangkok, Thailand; 9 Department of Microbiology, Faculty of Science, Mahidol University, Bangkok, Thailand; 10 Virology and Cell Technology Research Team, National Center for Genetic Engineering and Biotechnology (BIOTEC), National Science and Technology Development Agency (NSTDA), Pathum Thani, Thailand; 11 Department of Medicine, Faculty of Medicine, Chulalongkorn University, Bangkok, Thailand; 12 Clinical Research Laboratory/HIV-NAT Laboratory, ChulaCRC, Faculty of Medicine, Chulalongkorn University, Bangkok, Thailand; 13 HIVNAT, Thai Red Cross AIDS Research Centre, Bangkok, Thailand; 14 Biostatistics Excellence Centre, Faculty of Medicine, Chulalongkorn University, Bangkok, Thailand; 15 Biostatistics Unit, HIVNAT, Thai Red Cross AIDS Research Centre, Bangkok, Thailand; 16 The Kirby Institute, University of New South Wales, Sydney, Australia; 17 SEARCH Research Foundation, Bangkok, Thailand; 18 Department of Microbiology, Faculty of Medicine, Chulalongkorn University, Bangkok, Thailand; 19 Department of Pharmacology, Faculty of Medicine, Chulalongkorn University, Bangkok, Thailand; 20 Genevant Sciences Corporation, Vancouver, BC Canada; 21 Department of Surgery, Duke University Medical Center, Durham, NC USA; 22 BioNet Asia, Co. Ltd., Bangkok, Thailand; 23 Perelman School of Medicine, University of Pennsylvania, Philadelphia, PA USA; 24 Armed Forces Research Institute of Medical Sciences, Bangkok, Thailand

**Keywords:** Diseases, Infectious diseases, Vaccines, Medical research, Clinical trial design

## Abstract

ChulaCov19 mRNA vaccine demonstrated promising phase 1 results. Healthy adults aged 18–59 years were double-blind randomised 4:1 to receive two intramuscular doses of ChulaCov19 50 µg or placebo. Primary endpoints were safety and microneutralization antibody against-wild-type (Micro-VNT50) at day 50. One hundred fifty adults with median (IQR) age 37 (30–46) years were randomised. ChulaCov19 was well tolerated, and most adverse events were mild to moderate and temporary. Geometric mean titres (GMT) of neutralizing titre against wild-type for ChulaCov19 on day 50 were 1367 IU/mL. T-cell IFN-γ-ELISpot showed the highest responses at one week (Day29) after dose 2 then gradually declined. ChulaCov19 50 µg is well tolerated and elicited high neutralizing antibodies and strong T-cell responses in healthy adults.

**Trial registration number:** ClinicalTrials.gov Identifier NCT04566276, 28/09/2020.

## Introduction

During the SARS-CoV-2 pandemic, mRNA vaccine platforms were widely used since they can be rapidly produced to scale, and elicit high humoral and cell mediated immune responses^1^. As of 8 January 2023, more than thirteen billion doses of vaccine have been given globally. However, due to inequity in vaccine accessibility, only 25% of people in developing countries, in contrast to > 70% in the developed world, have received at least one dose of vaccine^2^. The ability to manufacture mRNA vaccines in low- and middle-income countries (LMICs) is important for this current, and future pandemics^3^. The ChulaCov19 mRNA vaccine (ChulaCov19) development program is an initiative of the Vaccine Research Center, Chulalongkorn University which aims to build capacity in development, manufacturing, clinical testing and regulatory filing of mRNA vaccines for pandemic preparedness, and therefore enhance timely access to vaccines in LMICs, now and in the future. The program is supported by the Thai Government and public donations. ChulaCov19 is a lipid nanoparticle (LNP) encapsulated nucleoside-modified mRNA encoding a prefusion non-stabilised, ectodomain of the SARS-CoV-2 spike protein of the original strain. ChulaCov19 differs from BNT162b2 (Comirnaty, Pfizer/BioNTech) and mRNA-1273 (Moderna) vaccines in two important ways: it is not prefusion stabilised, and it is encapsulated in a different LNP formula. Although long-tern storage stability of ChulaCov19 at − 75 ± 10 °C is at least 18 months, it is stable at 4–8 °C up to 3 months. In a phase 1 safety and dose ranging study, ChulaCov19 doses of 10, 25 and 50 μg were safe and well tolerated, and elicited robust humoral and cell mediated immunity in a dose-dependent and age-dependent manner, comparable to an approved mRNA COVID-19 vaccine^4^. Based on the safety profile and highest immunogenicity, ChulaCov19 50 μg was selected as the dose for further evaluation. This phase 2 study aimed to compare safety and immunogenicity of ChulaCov19 vaccine against a placebo, in healthy volunteers. The primary aim was to assess neutralizing titres against wild-type (WT) virus (Wuhan Hu-1 lineage). The secondary aim was to assess cross neutralization with other variants of concern. After 29 days, the randomized controlled study was unblinded, all participants in the placebo group received two doses of an approved mRNA vaccine for further immunogenicity comparisons as a post-hoc analysis. This paper presents the phase 2 study results.

## Methods

### Trial design

This phase 2 double-blind, placebo-controlled randomised study enrolled healthy adults aged 18–59 years. Inclusion and exclusion criteria are previously described elsewhere^4^ and shown in Supplemental Table S1. After enrolment, participants received two doses of 50 μg ChulaCov19 or placebo intramuscularly (IM), 21 days apart. At day 29 (1 week after Dose 2) which is the timepoint of immunogenicity primary endpoint analysis, the participant randomization code was unblinded. However, due to concern about participant safety during the Delta outbreak, the participants in placebo group received two doses of Comirnaty (Pfizer, USA, lot number 30125BA), 30 μg IM on days 29 and 50. Both groups continued study follow up until day 202 after the first vaccination.The trial and the Investigational New Drug application were approved by the ethics committee of the Faculty of Medicine, Chulalongkorn University, Bangkok (IRB number 833/63), and Thailand’s Food and Drug Administration, respectively. The study was conducted in accordance with the regulatory requirements of International Council for Harmonization Good Clinical Practice (GCP), the Consolidated Standards of Reporting Trials (CONSORT)^5^ and conformed to the principles Declaration of Helsinki. All participants provided written informed consent. The trial was conducted at the Chula Clinical Research Center and the King Chulalongkorn Memorial Hospital, Bangkok. ClinicalTrials.gov Identifier: NCT04566276, 28/09/2020.

### Trial vaccine

ChulaCov19 mRNA was manufactured at Trilink Biotechnologies (San Diego, California), and the vaccine was manufactured at Integrity Bio Inc. (Camarillo, California). The encapsulating LNP formulation consists of four lipid components with a proprietary ionised lipid developed by Genevant Sciences Corporation (Vancouver, British Columbia).ChulaCov19 was stored as a sterile 0.2 mg/mL suspension at − 75 ± 10 °C, then diluted with normal saline to 100 μg/mL and given as a 0.5 mL intramuscular injection. The placebo was prepared from 0.5 mL 0.9% sodium chloride. After the investigators enrolled a participant, injectable blinded trial medicines were prepared by the pharmacy, then sent to the clinic with the participant ID, and administered by the study nurse.

### Trial procedures

The vaccine was administered in the deltoid muscle on Day 1 and Day 22 ± 3. A diary was provided to participants to record solicited- and unsolicited adverse reactions, and concomitant medications, for seven days after each vaccination. Reactogenicity assessments included solicited and unsolicited local adverse events (AE), systemic AE, in the seven days after vaccination, and serious adverse events (SAE) up to Day 50 (four weeks after the second vaccination). Safety laboratory tests were performed at baseline, then days 8, 22, 29, 50. Study long term follow up visits occurred at days 112 and 202. During follow up, the number of participants who had COVID-19 infection were reported, as well as the clinical severity and treatment received. These participants were excluded from immunogenicity analyses from the time of infection.

### Assessment of immunogenicity

The primary immunogenicity endpoint was neutralizing antibody titres against wild-type virus (Wuhan Hu-1), assessed by the live-virus microneutralization assay (Micro-VNT50) at day 50. Secondary immunogenicity endpoints included (1)the proportion of participants who seroconverted (defined as achieving a ≥ fourfold rise in SARS-CoV-2-specific neutralizing antibodies and spike-protein binding IgG) at Day 29 versus baseline; (2) cellular immunity measured by IFNγ-ELISpot assay (ELISpot) at day 29; (3) cross neutralization to variants of concern, and Micro VNT-50 to variants of concern (Alpha (B.1.1.7), Beta (B.1.351), delta (B.1.617.2) variants) at day 29; (4) binding to the SARS-CoV-2 S1 receptor-binding domain (RBD) by enzyme-linked immunosorbent assay (ELISA) using SARS-CoV-2-RBD-specific IgG antibody (SARS-CoV-2 IgG II Quant) Chemiluminescent microparticle immunoassay (CMIA), (Architect, Abbott, Ireland); (5) SARS-CoV-2-specific serum neutralizing antibody results measured by pseudovirus neutralization test (psVNT50) against WT (Wuhan Hu-1), Delta (B.1.617.2) (performed at Biotec, NSTDA, Thailand), and Omicron variants (B.1.1.529) (performed at Department of Surgery, Duke University Medical Center, Durham, NC USA) at day 29 and day 50^6^. SARS-CoV-2 RBD-ACE2 blocking antibody was measured by surrogate viral neutralization test (sVNT). Participants initially randomised to placebo, who received Comirnaty, Pfizer/BioNTech mRNA vaccines on study days 29 and 50, had blood drawn for the primary immunogenicity endpoint comparison 28 days after the second Comirnaty vaccine dose as a post-hoc analysis. A follow up visit was scheduled at study day 202 (152 days after completing the 2-dose Comirnaty vaccination schedule).

### Statistical analysis

#### Sample size

Assuming no seroconversions would occur in the placebo arm and that the difference in seroconversion rates of Micro-VNT 50 in active vaccine participants compared to placebo participants would be 20%, with a 2-sided test at a 5% level of significance and 90% power using a 4:1 ratio, the calculated sample size for the vaccine:placebo groups required at least 116 active vaccine and 29 placebo participants, rounded up to 120 active vaccine and 30 placebo for implementation. The randomisation sequence was developed by an independent biostatistician using a block size of 5 with a 4:1 ratio of ChulaCov19 to placebo, and the pharmacy held the codes and assigned sequential codes to eligible participants.

#### Safety and immunogenicity

A diary was provided to participants to record solicited- and unsolicited adverse reactions, and concomitant medications for seven days. Haematological lab tests for safety including complete blood count, coagulation Prothrombin time (PT), partial thromboplastin time (PTT), and international normalised ratio (INR), and clinical chemistry lab tests including renal function, liver function, electrolyte, haemoglobin A1c, creatinine phosphokinase, calcium, uric acid, C-reactive protein, were performed at baseline days 8, 22, 29 and 50 (details in Supplemental Table S2).

Safety endpoints included solicited and unsolicited local adverse events (AE), systemic AE, use of antipyretics/analgesics in the seven days after vaccination, and serious adverse events (SAE) up to Day 50 (four weeks after second vaccination). Pain and tenderness at the injection site was graded as mild, moderate, or severe. Reactogenicity grading for was mild, moderate, severe, life-threatening or death as defined in the protocol. The frequency (%) of solicited and unsolicited specific local or systemic adverse events within seven days after administration of each dose of vaccine or placebo was summarised by severity grading. Other adverse events reported by participants who received at least one vaccine or placebo dose was described by study arm at day 29, with formal comparisons between ChulaCov19 and placebo, day 50 comparing ChulaCov19 and Comirnaty vaccine and until day 202. Formal comparisons of the proportion of participants experiencing any adverse events for the vaccine versus the placebo arm were made with a Chi-square or Fisher’s exact test as appropriate.

Immunogenicity was analysed based on the per protocol (PP) analysis set. Geometric mean titres (GMT) and their 95% CI were calculated for Micro-VNT50, anti RBD-IgG, IFNγ-ELISpot. Post-hoc analysis of Micro-VNT50, PsVNT50, anti RBD-IgG, and sVNT against samples of 30 participants in placebo arm who subsequently received Comirnaty vaccine, were made using linear regression models to calculate geometric mean titre ratios (GMTR 95%CI. Statistical analysis was conducted with Stata 15.1 (Statacorp LLC, College Station, TX, USA).

## Results

### Baseline characteristics

**Figure 1:**
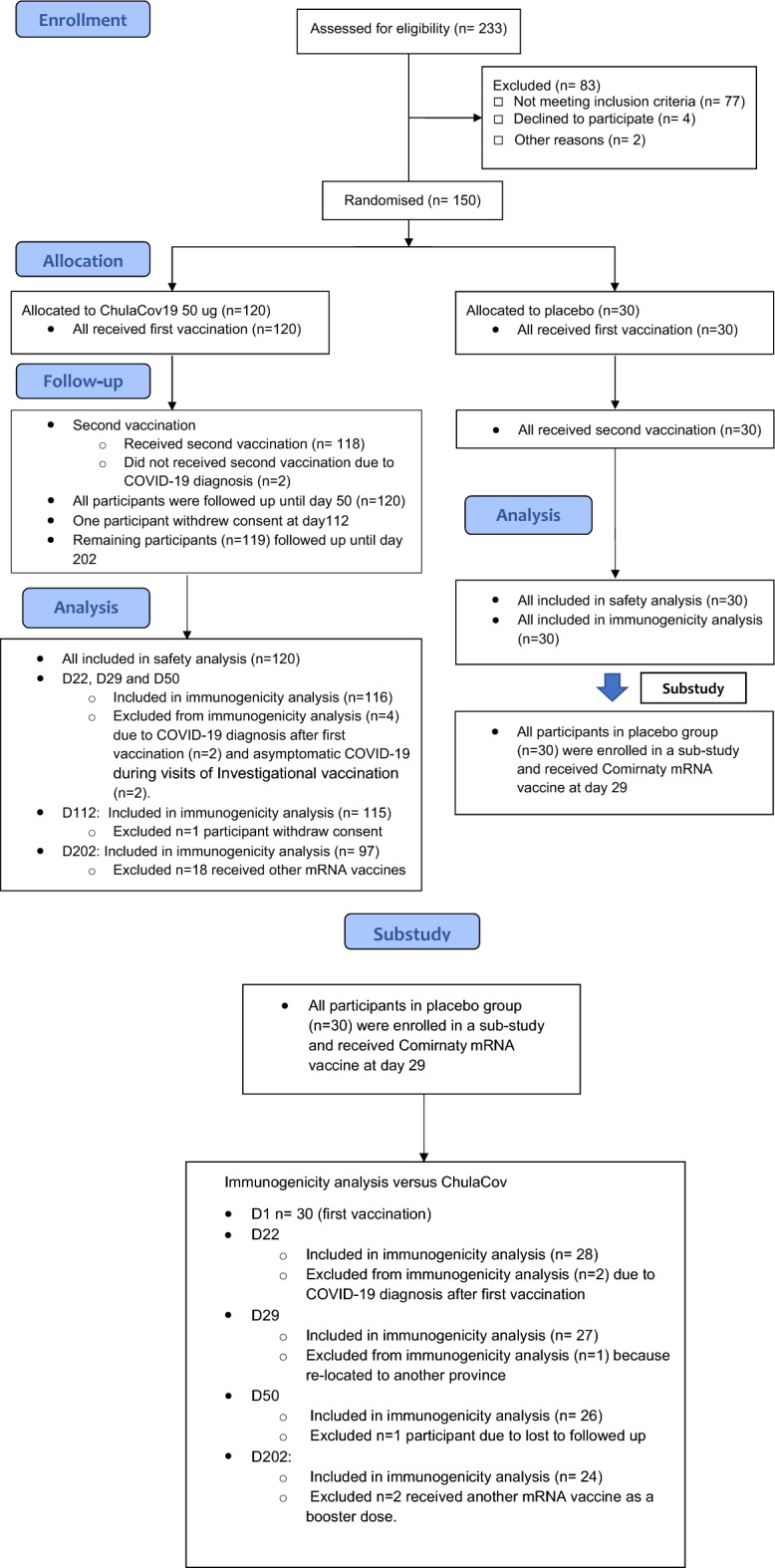
CONSORT 2010 study flow diagram.

Between 29 July to 3 August 2021, 233 individuals were screened, of whom 120 were enrolled to the ChulaCov19 group and 30 to the placebo group (Fig. 1). The median (interquartile range [IQR]) age in the vaccine and placebo groups was 38 (30–46) year and 37 (27–42) year, respectively. The proportion of males was 49% and 43%, respectively. Median body mass index (range) was 23.9 (18.4–30.0) and 23.6 (18.6–28.2) kg/m^2^, respectively. Study follow-up ceased on 17 March 2022 at day 202. In the main study which compared ChulaCov19 against placebo, 4/120 in the ChulaCov19 group and none of the placebo group were excluded from the immunogenicity analyses from day 22 onwards. Two infections in the ChulaCov group were asymptomatic and occurred prior to enrolment (anti-nucleocapsid positive), and two were acquired SARS-CoV-2 infections after the first vaccine dose. One participant withdrew consent and was excluded from the day 112 and 202 immunogenicity analyses, and an additional 18 participants who received an mRNA booster vaccine were excluded from the day 202 analysis. These early infections occurred during September 2021 when Delta variant was the dominant variant in circulation. In the sub-study comparing ChulaCov19 to Comirnaty, all placebo group participants received their first Comirnaty dose; two who acquired COVID-19 before their second dose were excluded from the day 22 immunogenicity comparison. One participant who moved to another province was excluded from the day 29 and day 50 immunogenicity analysis, and one who was lost was excluded from the day 50 immunogenicity analysis. Two participants who received another mRNA vaccine as a booster dose were excluded from the day 202 immunogenicity analysis. All participants contributed to the safety analysis.

### Safety and tolerability

**Figure 2:**
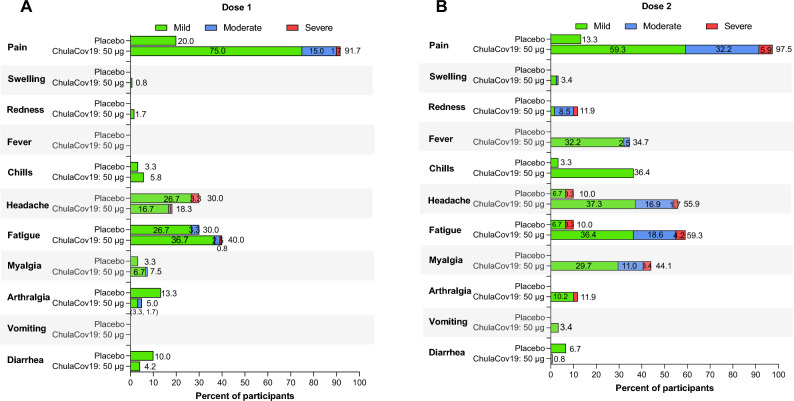
Solicited local and systemic reactions of ChulaCoV19 vaccine compared with placebo. (**A**) after first dose (Dose 1), (**B**) after second dose (Dose 2).

Data describing local and systemic solicited reactions are shown in Fig. 2A,B, Table 1 and Supplementary Table S3. The most common local reaction was injection site pain, which was mild (59.3%) and moderate (32.2%) after the second ChulaCov19 dose. No fever was observed after Dose 1; approximately one-third of participants had mild to moderate fever or chills after Dose 2. Approximately half of participants reported fatigue, headache, or myalgia after the second dose of vaccine, less than 5% of participants reported these reactions were severe. The adverse events were temporary with median (IQR) duration of local and systemic AEs 2 (2–3) and 1 (1–2) days, respectively.

**Table 1:** Overall study vaccine-related adverse events up to 50 days after the first vaccination.

Adverse event, N (%)	ChulaCov19, (N = 120) up to 50 days	Placebo, (N = 30) up to 29 days	Comirnaty (post-Placebo), (N = 30) up to 50 days
Overall adverse events	119 (99.2%)	26 (86.7%)	30 (100%)
Grade 3	16 (13.3%)	1 (3.3%)	0 (0.0)
Grade 4	0 (0.0)	0 (0.0)	0 (0.0)
Solicited adverse events			
All	118 (98.3%)	18 (60.0%)	30 (100%)
Grade 3	14 (11.7%)	1 (3.3%)	0 (0.0)
Grade 4	0 (0.0)	0 (0.0)	0 (0.0)
Unsolicited adverse events			
All	55 (45.8%)	11 (36.7%)	1 (3.3%)
Grade 3	2 (1.7%)	0 (0.0)	0 (0.0)
Grade 4	0 (0.0)	0 (0.0)	0 (0.0)
Serious adverse event	0 (0.0)	0 (0.0)	0 (0.0)

Higher rates of any AE were reported in the ChulaCov19 group (99%) than placebo recipients (87%). Grade 3 severity was reported by 16/120 (13.3%) ChulaCov19 versus 1/30 (3.3%) placebo recipients. Placebo-effects were common, after at least one dose, 30% of placebo arm participants reported headache, 30% reported fatigue and 10–13% reported myalgia, arthralgia, or diarrhea. During the study, non-vaccine-related SAE were reported in 30 participants (28/120 in the ChulaCov19 and 2/30 in the placebo/Comirnaty group). All were Covid-19 infections, 28 of mild and 2 of moderate severity, and unrelated to study drug.

Among 30 participants randomised to the placebo group who subsequently received Comirnaty vaccine, reported rates of systemic solicited reactions after second vaccine doses were lower than in the ChulaCov19 group: fatigue (3.7% vs 59.3%), headache (39.3% vs 55.9%), myalgia (17.9% vs 44.1%), respectively.

### Immunogenicity results

#### Live virus micro neutralization assay (Micro-VNT50)

**Figure 3:**
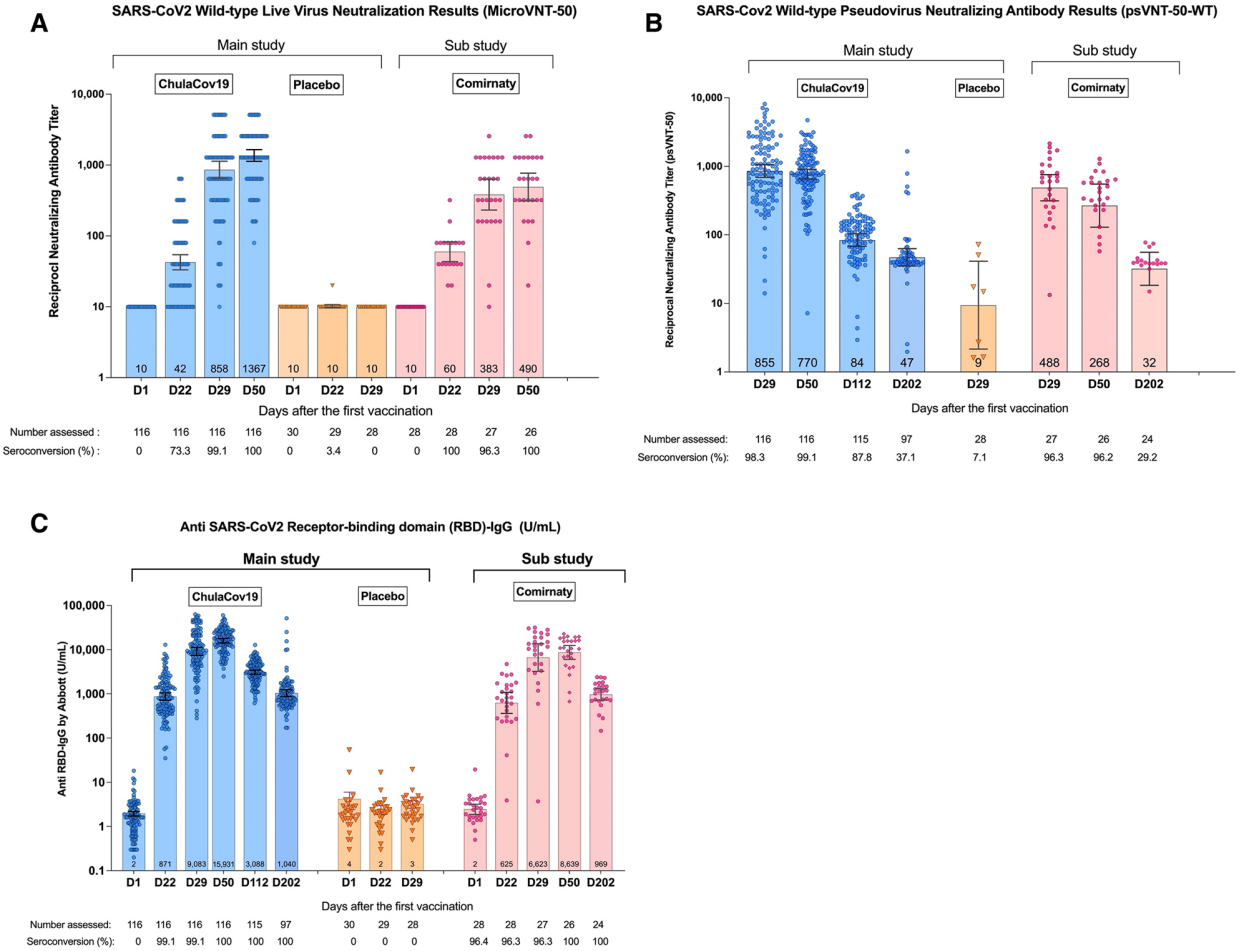
(**A**) Neutralizing antibody results measured by live virus microneutralization assay (Micro-VNT50) against SARS-CoV-2 wild type virus. Seroconversion defines as Micro-VNT50 titer ≥ 10. (**B**) SARS-Cov-2-specific serum neutralizing antibody titres measured by pseudovirus neutralization test (psVNT50). Seroconversion defines as psVNT-50 titer ≥ 10. (**C**) SARS-CoV2 receptor binding domain (RBD) antibody titres measured by ELISA. Seroconversion defines as anti-RBD-IgG ≥ 50 U/mL.

All participants in the per protocol study population had undetectable neutralizing antibody titres at baseline. Seroconversion occurred in all ChulaCov19 and one placebo group participant. GMT (95% CI) of the responses against WT virus among ChulaCov19 arm participants were 858 (645–1132) on day 29 and rose to 1367 (1131–1652) on day 50 (1 and 4 weeks after Dose 2, respectively). The participants in placebo arm who received Comirnaty had GMT of neutralizing antibodies against WT of 383 (232–633) on day 29 and 490 (313–768) on day 50 (Fig. 3A). These equate to GMTR of ChulaCov19/Comirnaty of 2.24 (95% CI 1.21–4.16) and 2.79 (95% CI 1.78–4.37) with p-values of 0.01 and 0.001, respectively (Supplemental Table S4). Progressive declines in neutralizing antibody titres were observed from the microneutralization assay against variants of concern, Alpha, Beta and Delta compared to WT, respectively as shown in Supplemental Table S4.

### Neutralizing antibody and its kinetics measured by pseudovirus microneutralization assay (psVNT50) against SARS-CoV-2 WT (Wuhan Hu-1), Delta (B.1.617.2), Omicron (B.1.1.529) variants of concern

**Figure 4:**
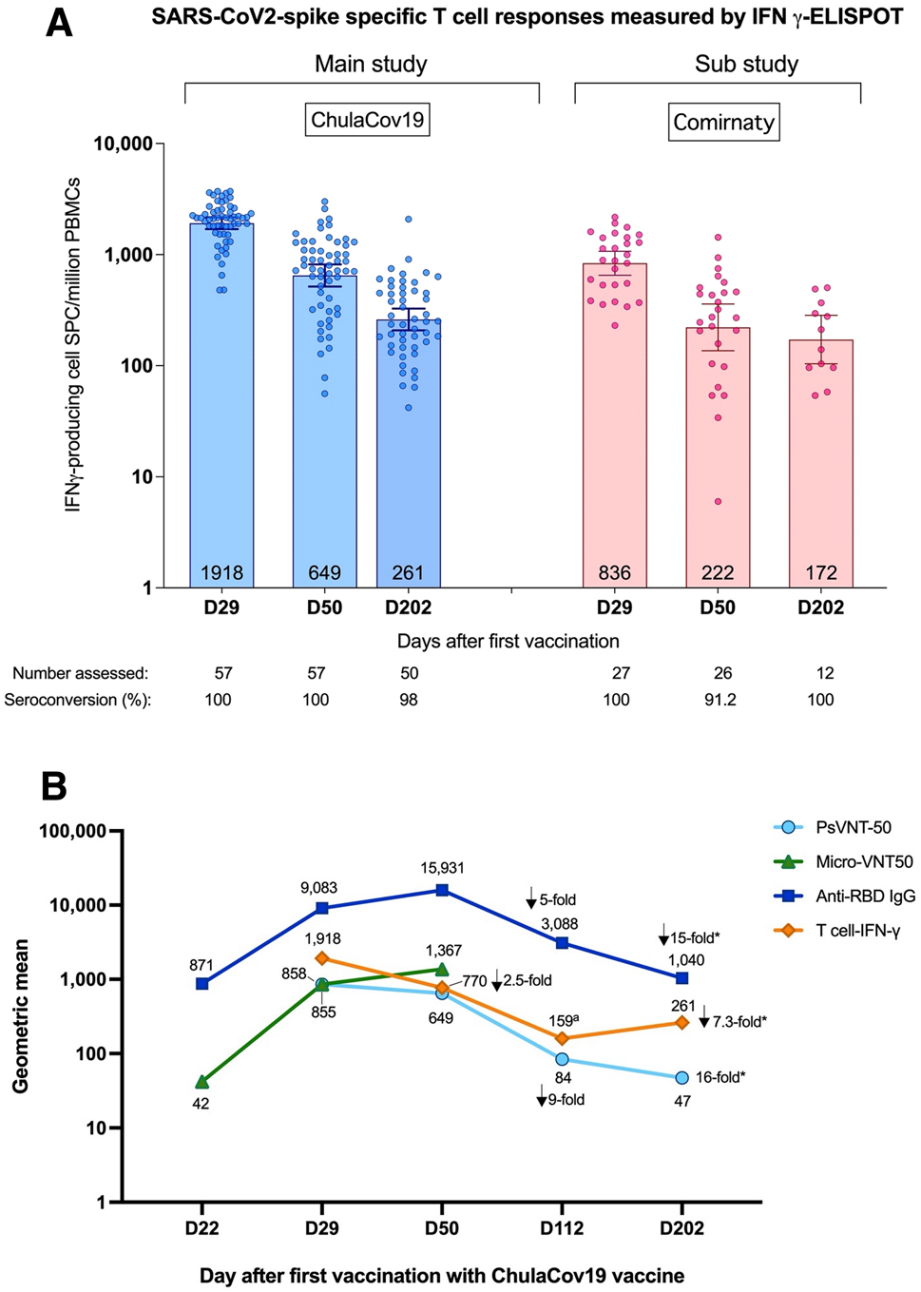
(**A**) SARS-Cov2 Wild-type Spike-specific T-cell responses measured by IFNγ-ELISpot assays. Seroconversion defines as spike-specific T cell ≥ 50 SCF/10^6^ PBMCs. (**B**) Kinetics of SARS-CoV2 Specific Antibody and T-cell Immune Responses. Antibodies (anti-RBD, Micro-VNT50 and psVNT50) are expressed as geometric mean titres, and of IFNγ-ELISpot T-cell responses are SPF/million PBMCs. *Indicates a decline in rate from peak; ^a^the T-cell response result at this timepoint was from frozen cells, all other timepoints were assayed using fresh PBMCs).

As shown in Fig. 3B, ChulaCov19 elicited GMT (95% CI) against WT of 855 (95% CI 690–1,058), 770 (95% CI 654–906), and 47 (95% CI 35–63); whereas GMT elicited by Comirnaty were 488 (95% CI 314–757), 268 (95% CI 130–552) and 32 (95% CI 18–56) at Days 29, 50 and 202, respectively. At Day 50, GMTR of ChulaCov19/ Comirnaty against WT virus was 2.88 (95% CI 1.79–4.62), p-value < 0.001, and against Delta variant was 1.77 (95% CI 1.15–2.72), p-value = 0.01 (Table 2). GMT of both ChulaCov19 and Comirnaty against Omicron variant showed a significant decline or > 30-fold at both Days 29 and 50 (Supplemental Fig. S1). Although the WT and Omicron psVNT50 assay were performed at Duke Medical Center, USA, and the results presented in Fig. 4 and Table 2 were performed at Biotec, NSTDA, Thailand, the GMT against WT results were consistent.

**Table 2:** Comparison of SARS-Cov-2-specific serum neutralizing antibody measured by pseudovirus neutralization test (psVNT50) against wild-type (WT) and delta variant between ChulaCov19 and comirnaty (Pfizer/BNT) vaccine.

psVNT50	Day	Vaccine	N	GMT			GMTR			P-value
				Value	95% CI		Value	95% CI		
					LL	UL		LL	UL	
WT, reciprocal titre	29	ChulaCov19	116	854.6	690.0	1058.4	1.75	1.07	2.86	0.03
		Comirnaty	27	487.5	314.0	757.0	Ref	Ref	Ref	Ref
	50	ChulaCov19	116	769.6	653.7	906.1	2.88	1.79	4.62	< 0.001
		Comirnaty	26	267.6	129.7	552.2	Ref	Ref	Ref	Ref
	202	ChulaCov19	97	47	35.1	62.8	1.46	0.80	2.68	0.21
		Comirnaty	24	32	18.4	55.8	Ref	Ref	Ref	Ref
Delta, reciprocal titre	29	ChulaCov19	116	324.2	234.1	449.0	1.21	0.58	2.52	0.62
		Comirnaty	27	268.9	206.4	350.3	Ref	Ref	Ref	Ref
	50	ChulaCov19	116	350.2	292.5	419.2	1.77	1.15	2.72	0.01
		Comirnaty	26	197.8	133.9	292.3	Ref	Ref	Ref	Ref

### SARS-Cov2 RBD antibody responses

The anti-RBD antibody was detectable 1 week after Dose 2 (Day 29), peaked 4 weeks after Dose 2 (Day 50), then declined 9-15-fold by Day202. This pattern was similar regardless of whether participants had been vaccinated with ChulaCov19 or Comirnaty. At Day 50, the GMTR of ChulaCov19/Comirnaty (GMT 15931/8639) was 1.84 (95% CI 1.38–2.47); p-value < 0.001 (Fig. 3C and Supplemental Table S5).

### Cell mediated immune response

As shown in Fig. 4A, at Day 29, all ChulaCov19 participants showed strong spike-specific T-cell responses measured by IFNγ-ELISpot tests, with GM (95% CI) of 1918 (1696–2168) SFC/million PBMCs, which subsequently declined to 649 (516–816) SFC/million PBMCs and 261 (208–327) SFC/million PBMCs at days 50 and 202 respectively. The T-cell responses among participants in placebo arm who subsequently received Comirnaty trended lower than those in the ChulaCov19 arm with GM (95% CI) of 836 (652–1072) SFC/million PBMCs on day 29, which declined to 222 (137–359) SFC/million PBMCs and 172 (104–284) SFC/million PBMCs at days 50 and 202 respectively. The GMTR of ChulaCov19/Comirnaty was 2.29 (95%CI 1.80–2.92) at Day 29 and 2.93 (95%CI 1.85–4.64) at Day 50 (p-value for both comparisons < 0.001), Supplemental Table S6.

### Overall kinetics of SARS-CoV2-specific antibody and T-cell immune responses

Figure 4B shows the geometric mean kinetics of specific immune responses. After Dose 2, T-cell responses showed the highest magnitude at one week (Day29) after immunization. The antibody responses peaked at one month after Dose 2 (Day 50). T-cell responses declined approximately one month earlier than antibodies. Neutralizing antibody measured by psVNT50 declined more rapidly compared to anti-RBD IgG antibody: by three months after Dose 2 (Day 112), the GMT of psVNT50 declined approximately nine-fold, whereas anti-RBD antibody declined five-fold. However, by six months (Day 202), the decline was fifteen-to-16-fold from peak levels for both psVNT50 and anti-RBD antibody. In contrast, T-cell responses declined only 7.3-fold at Day 202, compared to the peak response.

## Discussion

mRNA vaccines are the most effective vaccines against COVID19^7^. However, access to mRNA vaccines in low-and middle- income country settings remain limited. The main objective of the mRNA vaccine development program from which ChulaCov19 was derived, was to establish a complete ecosystem from the initial phases of research and development, through manufacturing, clinical development, and licensure. In doing so, current unmet capacity and capability of mRNA vaccine technology platforms would enable improved access to mRNA vaccines during this and future pandemics, in low-and middle-income countries. This ChulaCov19 phase 2 study confirms the recently published Phase 1 safety and immunogenicity results^4^. ChulaCov19 was well tolerated and elicited robust specific B-cell and T-cell responses.

In the ChulaCov19 arm, local injection site reactions were the most common adverse events, and systemic reactions were more common after Dose 2 than Dose 1. No fever was observed after Dose 1, but approximately one-third of participants reported fever after dose 2, of which most were mild (38.0–38.4 °C), and only 3/120 (2.5%) had moderate fever (38.5–38.9 °C). No grade 4 or serious adverse events were observed up to Day 50 of the study. Most local and systemic adverse events (AEs) were mild to moderate, and temporary, recovering within approximately two days. Of note, some AEs, particularly fatigue and headache can be placebo effects, and were observed in approximately 30–40% of placebo arm participants (Fig. 2 and Supplemental Table S3). In this study, the 30 placebo group participants received (Comirnaty (Pfizer/BNT) vaccine on Day29, so could serve as another control comparator group. The tolerability of two vaccines was similar, although ChulaCov19 was associated with more grade 3 AE than Comirnaty (Table 1). This is most likely related to the higher dosage of ChulaCov19 (50 μg/dose) versus Comirnaty (30 μg/dose). However, the smaller sample size in the Comirnaty arm (n = 30 versus n = 120) may bias this comparison. In the phase 3 efficacy report of Comirnaty, 16% of participants reported fever, of which approximately a third were moderate, and 0.2% were severe (38.9–40 °C)^6^. The percentages of fatigue, headache and myalgia observed post-vaccination were similar to those observed after ChulaCov19 **(**Fig. 1 and Supplemental Table S3).

ChulaCov19 elicited strong SARS-Cov2-specific T-cell and antibody responses. T-cell responses measured by IFNγ-ELISpot assay showed the highest level at 1 week after Dose 2, while the responses of all tested antibodies: sVNT (Supplemental Fig. S2), anti-RBD IgG antibody, Micro-VNT50 and psVNT-50, which peaked 4 weeks after Dose 2**.** The titres of psVNT50 declined up to two-fold more than anti-RBD antibody at 4 weeks after Dose 2 (Fig. 4B). Waning of Covid-19 vaccine-induced immunity is an expected phenomenon, and has shown a clear association with waning vaccine effectiveness^8^**.** Compared to Comirnaty, at one month after Dose 2, ChulaCov19 induced significantly higher IFNγ-ELISpot T-cells responses (2.9-fold), higher anti-RBD IgG GMT (1**.**84-fold), higher psVNT50 GMT (2.9-fold against WT, and 1.8 against Delta), and higher Micro-VNT-50 GMT (2.8-fold) (Table 2, Supplemental Tables S5, S6). Studies of immune protection against WT virus in Covid-19 vaccine studies have shown that neutralizing antibody and/or RBD-binding antibody levels, when calibrated with human convalescent sera, correlate with vaccine efficacy^9,10^. Comirnaty mRNA vaccine has consistently shown high levels of protection against COVID-19 disease. Although the role of T-cell immunity in COVID19 vaccine efficacy is uncertain, its role in preventing transmission and reducing disease severity have been proven in animal model^11^. The other approved mRNA vaccine is mRNA-1273 (Moderna). Their phase 2 study compared two doses of 50 μg and 100 μg^12^. It is interesting that the magnitude of the live virus neutralizing antibody GMT observed after ChulaCov19 at 50 μg in this report, is similar to the GMT induced by both the 50 and 100 μg doses of mRNA-1273. This supports that based on our phase 2 results, ChulaCov19 is a promising candidate for further late clinical development.

Cross-neutralization against variants of concern is limited by two-dose regimens, as observed by responses of ChulaCov19 and Comirnaty to Omicron in particular (Supplemental Fig. S1). To improve vaccine effectiveness against variants of concern, particularly Delta and Omicron, administering mRNA vaccine booster doses has led to strong protection against both Delta and Omicron Covid-19-related hospitalization and death: this strategy was highly effective against symptomatic Delta but less effective against symptomatic Omicron infection^13,14^. The World Health Organization recommendations therefore support vaccine boosting against variants of concern^15^. Nonetheless, optimal intervals and frequency of vaccine boosting have not yet been fully studied and boosting every 4 to 6 months is not a practical long-term strategy. Robustly designed studies are required to inform evidence-based boosting strategies.

ChulaCov19 vaccine has 2 major differences from both approved mRNA vaccines. First, the mRNA sequence of ChulaCov19 encodes only for the extracellular domain and not the cytoplasmic domain of SARS-CoV2 spike protein, and the design does not include the di-proline mutation. This means the translated spike protein is secreted as a prefusion-non-stabilised form. In contrast, both approved mRNA vaccines consist of full-length spike sequences with two proline substitutions (K986P/V987P) that generate membrane-bound, prefusion-stabilised spike protein. Second, the lipid nanoparticle formulation for encapsulation of ChulaCov19 is a proprietary product of Genevant Sciences Corporation, whereas of the formulation used for Comirnaty is made by Acuitas Therapeutics, and Moderna developed their own propriety formulation for mRNA1273. In addition, as reported in our phase 1 ChulaCov19 study, ChulaCov19 is stable at 2–8 °C for up to 3 months^4^. These phase 2 results have further confirmed the findings of our phase 1 study, that ChulaCov19 elicited robust and strong specific immunogenicity like those of the approved mRNA vaccines, despite its differences^6,12^.Local manufacturing capacity has been established in partnership with BioNet Asia, Thailand, and a clinical lot produced in Thailand is undergoing further clinical trials in Thailand and Australia^16,17^. There are limitations of this study. First, the objective of the study was to assess immunogenicity and safety of mRNA vaccine against wild-type at day 50, but we excluded 4 participants who had asymptomatic and symptomatic COVID19 infections from immunogenicity endpoint due to interference from natural infection. This may have potential bias towards higher reported GMT of neutralization antibody, if the assumption was participants who acquired natural infection may have lower immunogenicity response compared to the other participants in the immunogenicity cohort. Secondly, due the surge in the Omicron variant and its sub-lineage, first-generation vaccines are no longer relevant. New generation bivalent (WT/Omicron) or monovalent XBB.1.5 sub-lineage mRNA vaccines have recently been approved in some countries in 2023. We have therefore developed a newer generation of mRNA vaccine targeting the most recent circulating viruses including XBB.1.5, XBB.1.16, and EG.5.1.

In summary, the ChulaCov19 mRNA vaccine development program aimed to establish capacity in low-and middle- income countries to improve timely and wider access to mRNA vaccines during pandemics. ChulaCov19, a secreted prefusion non-stabilised spike mRNA vaccine, at 50 μg is safe and well tolerated, and elicits robust and strong specific binding neutralizing antibodies, as well as T-cell immune responses similar to those elicited by currently approved mRNA vaccines. A clinical lot manufactured in Thailand is currently undergoing clinical trials in Thailand and Australia, and a new generation bivalent vaccine has been produced and will begin clinical evaluation in next few months.

### Supplementary Information

Supplementary Information 1.

Supplementary Information 2.

Supplementary Information 3.

Supplementary Information 4.

Supplementary Information 5.

## Data Availability

All data generated or analysed during this study are included in this published article and its supplementary information files.
