# Supplementary material for: Phase II prefusion non-stabilised Covid-19 mRNA vaccine randomised study
Source: Sci Rep. 2024 Jan 29;14:2373. doi: 10.1038/s41598-023-49653-6 (PMC10825165; doi:10.1038/s41598-023-49653-6)

**Supplementary Appendix**

This appendix has been provided by the authors to give readers additional information about their work.

Supplement to: Thanyawee, P. et al. Phase II prefusion non-stabilised Covid-19 mRNA vaccine randomised study

**Table of contents:**

| **Supplementary Appendix** | **Page** |
| --- | --- |
| 1. Supplement Table S1: Inclusion and exclusion criteria | 3-7 |
| 1. Supplement Table S2: Schedule for Safety Assessments | 8 |
| 1. Supplement Table S3 Solicited local and systematic reaction in participants who received ChulaCoV 19 versus placebo. | 9-10 |
| 1. Supplemental Table S4 Comparison of SARS-CoV-2 serum neutralizing antibody measured by live virus microneutralization assay (Micro VNT50) of wild type, alpha, beta and delta variants in participants who receive ChulaCoV19 vaccine and Comirnaty vaccine (Pfizer/BioNTech) | 11 |
| 1. Supplemental Table S5. Comparison of anti-SARS-CoV-2-RBD IgG antibody results between ChulaCov19 and Comirnaty (Pfizer/BioNTech) randomised groups | 12 |
| 1. Supplemental Table S6. SARS-CoV-2 Spike-specific T-Cell Responses measured by IFN γ-ELISPOT between ChulaCOV19 and Comirnaty (Pfizer/BioNTech) randomised groups. | 13 |
| 1. Supplemental Table S7 Comparison of % inhibition by sVNT between ChulaCov19 vaccine 50 µg and Comirnaty, Pfizer/BNT 30 µg | 14 |
| 1. Supplemental Figure S1: Pseudovirus neutralizing antibody (psVNT50) results against Omicron variant (performed at Duke University Medical Center, Durham, NC USA) (Comirnaty is Comirnaty, Pfizer/BioNTech). | 15 |
| 1. Supplemental Figure S2: Results of the SARS-CoV-2 RBD-ACE2 blocking antibody (% binding inhibition was measured by surrogate viral neutralization test (sVNT). | 16 |

**Supplement Table S1:** Inclusion and exclusion criteria

| **Inclusion Criteria**  Participants who met all the following criteria at screening were eligible to participate in the study:   1. Participants must be able to communicate effectively with study personnel and considered reliable, willing, and cooperative in terms of compliance with the protocol requirements. 2. Participants must sign the written informed consent form prior to undertaking any protocol related procedures. 3. Participants must have a body mass index (BMI) at Screening, calculated as the body mass divided (in kilograms [kg]) by the square of the body height (in meters [m]) of 18.0-30.0 kg/m^2^, inclusive. 4. Participants must have haematology (haemoglobin (Hb), haematocrit (Hct), red blood cell (RBC) and RBC morphology, white blood cell (WBC) and differential white blood cell count, platelet count (PLTs)), clinical chemistry (Sodium, potassium, chloride, bicarbonate, Blood Urea Nitrogen (BUN), creatinine, total protein, albumin, lipase, phosphorus, gamma-glutamyl transferase (GGT), hemoglobin A1c, creatinine phosphokinase (CPK), calcium, uric acid, C-reactive protein (CRP), alanine transaminase (ALT), aspartate transaminase (AST), creatinine (Cr), alkaline phosphatase (ALP), total bilirubin (T. Bili), estimated glomerular filtration rate (eGFR)), coagulation (Prothrombin time (PT), and partial thromboplastin time (PTT), international normalised ratio (INR)), urinalysis test results that are not deviating from the normal reference range by age and gender to a clinically relevant extent at Screening. 5. Males must be surgically sterile (>30 days since vasectomy with no viable sperm), practice true abstinence or, if engaged in sexual relations with a female of child-bearing potential, the participants and their partner must use an acceptable, highly effective, double-barrier contraceptive method* from Screening and for a period of at least 60 days after the last dose of investigational vaccine. 6. Women of child-bearing potential must practice true abstinence or, if engaged in sexual relations with a male, they must agree to use highly effective (failure rate of < 1% per year when used consistently and correctly), double-barrier contraceptive measures* throughout the study and intend to continue use of contraception for at least 60 days following the last vaccination.   * The PI assessed the adequacy of methods of contraception on a case-by-case basis. These criteria did not apply if the participants were in a same-sex relationship.   1. Women of child-bearing potential must have a negative serum pregnancy test (beta human chorionic gonadotropin [β-HCG]) at Screening and a negative urine-based test within 24 hours prior to each investigational vaccine administration. 2. Women of non-child-bearing potential must:  - be classified as being postmenopausal (defined as having a history of amenorrhea of at least one year), or - where history of amenorrhea is less than one-year, female participants must have an FSH level > 40 milli-international units per millilitre (mIU/mL), or - have a documented status of being surgically sterile (hysterectomy, bilateral oophorectomy, or tubal ligation/ salpingectomy).  1. Participants must be in general good health based on medical history and physical examination, as determined by the PI at Screening. 2. Body temperature must be less than 37.8ºC at Screening. 3. Pulse must be no greater than 100 beats per minute at Screening. 4. Systolic blood pressure (SBP) must be between 85 to 150 millimetres of mercury (mm Hg), inclusive at Screening. 5. Participants must agree to refrain from donating blood, plasma, ovules, sperm, or organs during the whole study. 6. Must be a male or female aged 18 - 59 (inclusive) at the time of enrolment.   **Exclusion Criteria**  Participants who met any of the following criteria were not eligible to participate in the study:   1. Presence of clinically significant medical history, unstable chronic or acute disease, or physical, or laboratory findings that, in the opinion of the PI may potentially increase the expected risk of exposure to the investigational vaccine, compromise the safety of the participant, or interfere with any aspect of study conduct or interpretation of results. This will include asthma and any thrombocytopenia or bleeding disorder contraindicating IM vaccination. 2. Presence of self-reported or medically documented significant medical or psychiatric condition(s). 3. Presence of an acute illness, as determined by the participating site PI or appropriate sub-PI, with or without fever (temperature≥ 38.0 ºC) within 72 hours prior to each vaccination. 4. Presence of birthmarks, tattoos, wound, or other skin conditions over the deltoid region of both arms that, in the PI’s opinion, could reasonably obscure and interfere with evaluation of local ISRs. 5. Inadequate venous access to allow collection of blood samples. 6. Breastfeeding or planning to breastfeed from the time of the first vaccination through 60 days after the last vaccination, or pregnant as confirmed by a positive serum β-HCG pregnancy test at Screening or positive urine pregnancy test at subsequent clinic visits at timepoints as delineated in the schedule of assessments. 7. Received any prophylactic or therapeutic vaccine, or licensed or unlicensed vaccine, drug, biologic, device, blood product, or medication, within 4 weeks of first vaccination or 5 half-lives (whichever is longer), or anticipate to do so in the follow-up period defined for this study. 8. Participant has previously participated in an investigational study involving LNPs (a component of the investigational vaccine assessed in this trial). 9. History of severe allergy (requiring hospital care), severe reaction to any drug or prior vaccination, or any known or suspected allergies or sensitivities to any component of the investigational vaccine or placebo. 10. History of ever had an anaphylaxis reaction to food, medication or vaccination. 11. Participant is immunosuppressed as caused by disease (such as HIV). 12. Chronic use (more than 14 continuous days) of or anticipated need to use, within the next 6 months of any medications that may be associated with impaired immune responsiveness or with immunosuppression. 13. History of hepatitis B or hepatitis C infection. 14. Receipt of immunoglobulins or blood products within 3 months of first vaccination. 15. Requirement for antipyretic or analgesic medication on a daily or every other day basis from enrolment through 72 hours after vaccination. 16. Current use of any prescription or over-the-counter medications within 7 days prior to vaccination, unless approved by the PI. 17. History of alcohol or drug abuse that in the opinion of the PI could affect the participant’s safety or compliance with study. 18. Participant unwilling to abstain from blood donation during the course of the study, and/or participation in any research study involving blood sampling (more than 450 mL /unit of blood), or blood donation to any blood bank during the 2 months prior to the Screening visit. 19. Close contact with anyone known to have SARS-CoV-2 infection within 30 days prior to vaccine administration. 20. Positive on SAR-CoV-2 antibody IgG/ IgM at screening 21. History of COVID-19 diagnosis (the criteria for COVID-19 diagnosis will follow the local guidelines). 22. On current treatment with investigational agents for prophylaxis of COVID-19. 23. Planning to travel outside Thailand from enrolment through 28 days after the second vaccination. 24. Residing in a nursing home or other skilled nursing facility or having a requirement for skilled nursing care. 25. Is a participant at high risk of SARS-CoV-2 exposure in the opinion of the PI (e.g., healthcare workers, active health care workers with direct patient contact, emergency response personnel). 26. Presence of co-morbidities that can be associated with an increased risk of severe COVID-19 Cancer, Chronic kidney diseases, COPD, cardiovascular disease, solid organ transplantation, DM type 2, uncontrolled HT, cerebrovascular disease |
| --- |

**Supplement Table S2: Schedule for Safety Assessments**

| **Assessments** | **Screening** | **Study treatment period** | | | **Follow-up** | |
| --- | --- | --- | --- | --- | --- | --- |
|  | **From Day -42** | **Day 1 (Baseline)** | **Day 8 (±3)** | **Day 22 (±3)** | **Day 29 (±3)** | **Day 50 (±3)** |
| Chemistry | 🗸 | 🗸 | 🗸 | 🗸 | 🗸 | 🗸 |
| Haematology | 🗸 | 🗸 | 🗸 | 🗸 | 🗸 | 🗸 |
| Coagulation | 🗸 | 🗸 | 🗸 | 🗸 | 🗸 | 🗸 |
| Urinalysis | 🗸 | 🗸 | 🗸 | 🗸 | 🗸 | 🗸 |
| Review diary booklet |  |  | 🗸 |  | 🗸 |  |

**Supplement Table S3:** Solicited local and systemic reactions in participants who received ChulaCoV19 versus placebo.

| AE | Severity | Dose 1 | | Dose 2 | |
| --- | --- | --- | --- | --- | --- |
|  |  | ChulaCov19: 50 µg  N=120 | Placebo  N=30 | ChulaCov19: 50 µg  N=118 | Placebo  N=30 |
| Solicited local reaction | | | | | |
| Injection site pain | Any | 110 (91.7%) | 6 (20%) | 115 (97.5%) | 4 (13.3%) |
|  | Mild | 90 (75%) | 6 (20%) | 70 (59.3%) | 4 (13.3%) |
|  | Moderate | 18 (15%) | 0 | 38 (32.2%) | 0 |
|  | Severe | 2 (1.7%) | 0 | 7 (5.9%) | 0 |
| Swelling | Any | 1 (0.8%) | 0 | 4 (3.4%) | 0 |
|  | Mild | 1 (0.8%) | 0 | 3 (2.55) | 0 |
|  | Moderate | 0 | 0 | 1 (0.8%) | 0 |
|  | Severe | 0 | 0 | 0 | 0 |
| Redness | Any | 2 (1.7%) | 0 | 14 (11.9%) | 0 |
|  | Mild | 2 (1.7%) | 0 | 2 (1.7%) | 0 |
|  | Moderate | 0 | 0 | 10 (8.5%) | 0 |
|  | Severe | 0 | 0 | 2 (1.7%) | 0 |
| Solicited systemic reaction | | | | | |
| Fever | Any | 0 | 0 | 41 (34.7%) | 0 |
|  | Mild | 0 | 0 | 38 (32.2%) | 0 |
|  | Moderate | 0 | 0 | 3 (2.5%) | 0 |
|  | Severe | 0 | 0 | 0 | 0 |
| Chills | Any | 7 (5.8%) | 1 (3.3%) | 43 (36.4%) | 1 (3.3%) |
|  | Mild | 7 (5.8%) | 1 (3.3%) | 43 (36.4%) | 1 (3.3%) |
| Headache | Any | 22 (18.3%) | 9 (30%) | 66 (55.9%) | 3 (10%) |
|  | Mild | 20 (16.7%) | 8 (26.7) | 44 (37.3%) | 2 (6.7%) |
|  | Moderate | 1 (0.8%) | 0 | 20 (16.9%) | 0 |
|  | Severe | 1 (0.8) | 1 (3.3%) | 2 (1.7%) | 1 (3.3%) |
| Fatigue | Any | 48 (40%) | 9 (30%) | 70 (59.3%) | 3 (10%) |
|  | Mild | 44 (36.7%) | 8 (26.7%) | 43 (36.4%) | 2 (6.7%) |
|  | Moderate | 3 (2.5%) | 1 (3.3%) | 22 (18.6%) | 0 |
|  | Severe | 1 (0.8%) | 0 | 5 (4.2%) | 1 (3.3%) |
| Myalgia | Any | 9 (7.5%) | 1 (3.3%) | 52 (44.1%) | 0 |
|  | Mild | 8 (6.7%) | 1 (3.3%) | 35 (29.7%) | 0 |
|  | Moderate | 1 (0.8%) | 0 | 13 (11%) | 0 |
|  | Severe | 0 | 0 | 4 (3.4%) | 0 |
| Arthralgia | Any | 6 (5%) | 4 (13.3%) | 14 (11.9%) | 0 |
|  | Mild | 4 (3.3%) | 4 (13.3%) | 12 (10.2%) | 0 |
|  | Moderate | 2 (1.7%) | 0 | 0 | 0 |
|  | Severe | 0 | 0 | 2 (1.7%) | 0 |
| Vomiting | Any | 0 | 0 | 4 (3.4%) | 0 |
|  | Mild | 0 | 0 | 4 (3.4%) | 0 |
| Diarrhea | Any | 5 (4.2%) | 3 (10%) | 1 (0.8%) | 2 (6.7%) |
|  | Mild | 5 (4.2%) | 3 (10%) | 1 (0.8%) | 2 (6.7%) |
|  | Moderate | 0 | 0 | 0 | 0 |
|  | Severe | 0 | 0 | 0 | 0 |

**Supplemental Table S4:** Comparison of SARS-CoV-2 serum neutralizing antibody measured by live virus microneutralization assay (Micro-VNT50) of wild type, alpha, beta and delta variants in participants who receive ChulaCoV19 vaccine and Comirnaty (Pfizer/BioNTech)

| Micro  VNT50 | Day | Vaccine | N | GMT | | | GMTR | | | P-value |
| --- | --- | --- | --- | --- | --- | --- | --- | --- | --- | --- |
|  |  |  |  | **microVNT50** | **95%CI** | | **Ratio** | **95%CI** | |  |
|  |  |  |  |  | **LL** | **UL** |  | **LL** | **UL** |  |
| WT | **29** | ChulaCov19 | 116 | 857.7 | 645 | 1131.8 | 2.24 | 1.21 | 4.16 | 0.01 |
|  |  | Comirnaty | 27 | 383 | 231.8 | 632.8 | Ref | Ref | Ref | Ref |
|  | **50** | ChulaCov19 | 116 | 1367 | 1131 | 1652 | 2.79 | 1.78 | 4.37 | <0.001 |
|  |  | Comirnaty | 26 | 490.2 | 313 | 767.8 | Ref | Ref | Ref | Ref |
| Alpha | **29** | ChulaCov19 | 116 | 255.5 | 195.7 | 333.7 | 0.96 | 0.52 | 1.75 | 0.88 |
|  |  | Comirnaty | 27 | 267.4 | 155.7 | 459.2 | Ref | Ref | Ref | Ref |
| Beta | **29** | ChulaCov19 | 116 | 70.0 | 54.8 | 89.3 | 0.66 | 0.38 | 1.15 | 0.14 |
|  |  | Comirnaty | 27 | 106.1 | 61.9 | 181.9 | Ref | Ref | Ref | Ref |
| Delta | **29** | ChulaCov19 | 116 | 56.6 | 45.3 | 70.7 | 1.01 | 0.61 | 1.68 | 0.96 |
|  |  | Comirnaty | 27 | 55.8 | 34.7 | 90.0 | Ref | Ref | Ref | Ref |
| GMT: Geometric mean titre, GMTR: Geometric mean titre ratio, 95%CI: 95% confidence interval, LL = lower limit, UL = upper limit, P-value were evaluated by Two-sample independent t-test, ref: reference | | | | | | | | | | |

Note: Excluded 4 ChulaCov19 participants: RA119 (baseline anti-N positive, RA002 (symptomatic COVID on day 11), RA013 (symptomatic COVID on day 16), and RA130 (anti-N+ on day 22); and 2 Placebo participants: RA004 (COVID on day 32) RA070 (COVID on day 2).

**Supplemental Table S5.** Comparison of anti-SARS-CoV-2-RBD IgG antibody results between ChulaCov19 and Comirnaty (Pfizer/BioNTech) randomised groups

| Day | Vaccine | N | GMT | | | GMTR | | | P-value |
| --- | --- | --- | --- | --- | --- | --- | --- | --- | --- |
|  |  |  | U/mL | 95%CI | | Ratio | 95%CI | |  |
|  |  |  |  | LL | UL |  | LL | UL |  |
| 29 | ChulaCov19 | 116 | 9083 | 7378 | 11180 | 1.37 | 0.80 | 2.36 | 0.25 |
|  | Comirnaty | 27 | 6623 | 3230 | 13583 | Ref | Ref | Ref | Ref |
| 50 | ChulaCov19 | 116 | 15931 | 14211 | 17859 | 1.84 | 1.38 | 2.47 | <0.001 |
|  | Comirnaty | 26 | 8639 | 6009 | 12420 | Ref | Ref | Ref | Ref |
| 112 | ChulaCov19 | 115 | 3088 | 2739 | 3398 | NA | NA | NA | NA |
| 202 | ChulaCov19 | 97 | 1040 | 873 | 1242 | 1.07 | 0.73 | 1.58 | 0.72 |
|  | Comirnaty | 24 | 969 | 721 | 1302 | Ref | Ref | Ref | Ref |
| GMT: Geometric mean titre, GMTR: Geometric mean titre ratio, 95%CI: 95% confidence interval, LL = lower limit, UL = upper limit, P-value were evaluated by Two-sample independent t-test, ref: reference | | | | | | | | | |

**Supplemental Table S6.** SARS-CoV-2 Spike-specific T-Cell Responses measured by IFN γ-ELISPOT between ChulaCOV19 and Comirnaty (Pfizer/BioNTech) randomised groups.

| Day | Vaccine | N | GM | | | | GMR | | | P-value |
| --- | --- | --- | --- | --- | --- | --- | --- | --- | --- | --- |
|  |  |  | **SFC/10^6^**  **PBMCs** | **95%CI** | | **Value** | | **95%CI** | |  |
|  |  |  |  | **LL** | **UL** |  |  | **LL** | **UL** |  |
| 29 | ChulaCov19 | 57 | 1918.0 | 1696.3 | 2168.7 | 2.29 | | 1.80 | 2.92 | <0.001 |
|  | Comirnaty | 27 | 836.1 | 652.0 | 1072.1 | Ref | | Ref | Ref | Ref |
| 50 | ChulaCov19 | 57 | 648.7 | 515.9 | 815.8 | 2.93 | | 1.85 | 4.64 | <0.001 |
|  | Comirnaty | 26 | 221.5 | 136.8 | 358.8 | Ref | | Ref | Ref | Ref |
| 202 | ChulaCov19 | 50 | 260.9 | 208.3 | 326.8 | 1.52 | | 0.91 | 2.53 | 0.11 |
|  | Comirnaty | 12 | 171.9 | 103.9 | 284.3 | Ref | | Ref | Ref | Ref |
| GMT: Geometric mean titre, GMFR: Geometric mean titre ratio, 95%CI: 95% confidence interval, LL = lower limit, UL = upper limit, P-value were evaluated by Two-sample independent t-test, ref: reference, | | | | | | | | | | |

**Supplemental Table S7:** Comparison of % inhibition by sVNT between ChulaCov19 vaccine 50 µg and Comirnaty (Pfizer/BNT 30 µg)

| Day | Vaccine | N | GM | | | GMR | | | P-value |
| --- | --- | --- | --- | --- | --- | --- | --- | --- | --- |
|  |  |  | Value | 95%CI | | Value | 95%CI | |  |
|  |  |  |  | LL | UL |  | LL | UL |  |
| 29 | ChulaCov 50 µg | 116 | 92.8 | 91.0 | 94.5 | 1.04 | 0.98 | 1.10 | 0.24 |
|  | Comirnaty | 27 | 89.2 | 81.1 | 98.2 | Ref | Ref | Ref | Ref |
| 50 | ChulaCov 50 µg | 116 | 95.8 | 95.3 | 96.2 | 1.03 | 1.01 | 1.05 | 0.01 |
|  | Comirnaty | 26 | 93.1 | 89.5 | 97.0 | Ref | Ref | Ref | Ref |
| 112 | ChulaCov 50 µg | 115 | 90.7 | 89.0 | 92.6 | na | na | na | na |
| 202 | ChulaCov 50 µg | 97 | 74.6 | 70.4 | 79.0 | 1.01 | 0.88 | 1.16 | 0.84 |
|  | Comirnaty | 24 | 73.6 | 62.8 | 86.1 | Ref | Ref | Ref | Ref |

GM: Geometric mean, GMR: Geometric mean ratio, 95%CI: 95% confidence interval, LL = lower limit, UL = upper limit, P-value were evaluated by Two-sample independent t-test, ref: reference, na = not applicable

**Supplemental figure S1**: Pseudovirus neutralizing antibody (psVNT50) results against Omicron variant (performed at Duke University Medical Center, Durham, NC USA) (Comirnaty is Comirnaty, Pfizer/BioNTech).


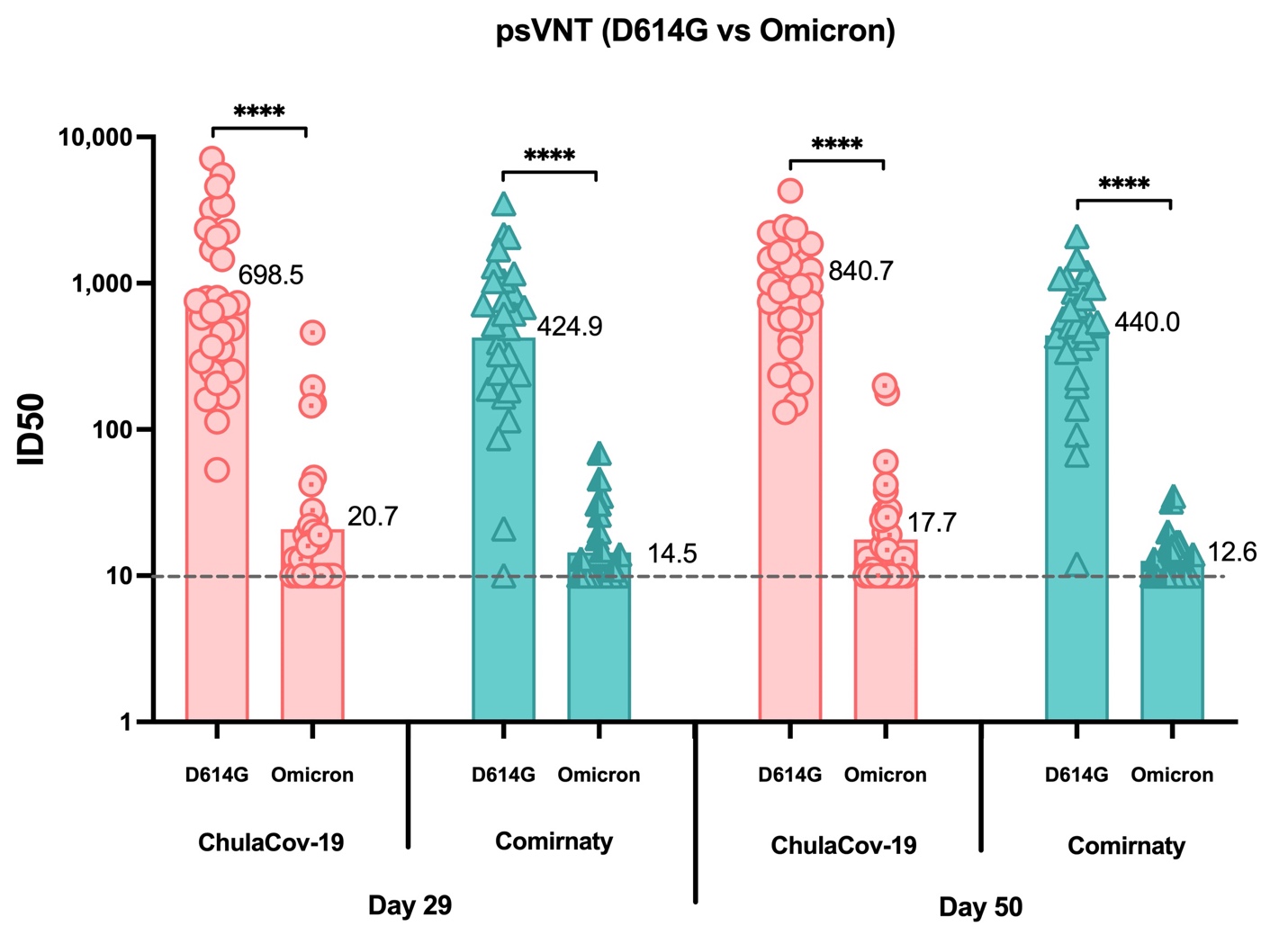


**Supplemental Figure S2:** Results of the SARS-CoV-2 RBD-ACE2 blocking antibody (% binding inhibition) was measured by surrogate viral neutralization test (sVNT). sVNT seroconversion defined as % inhibition >30%


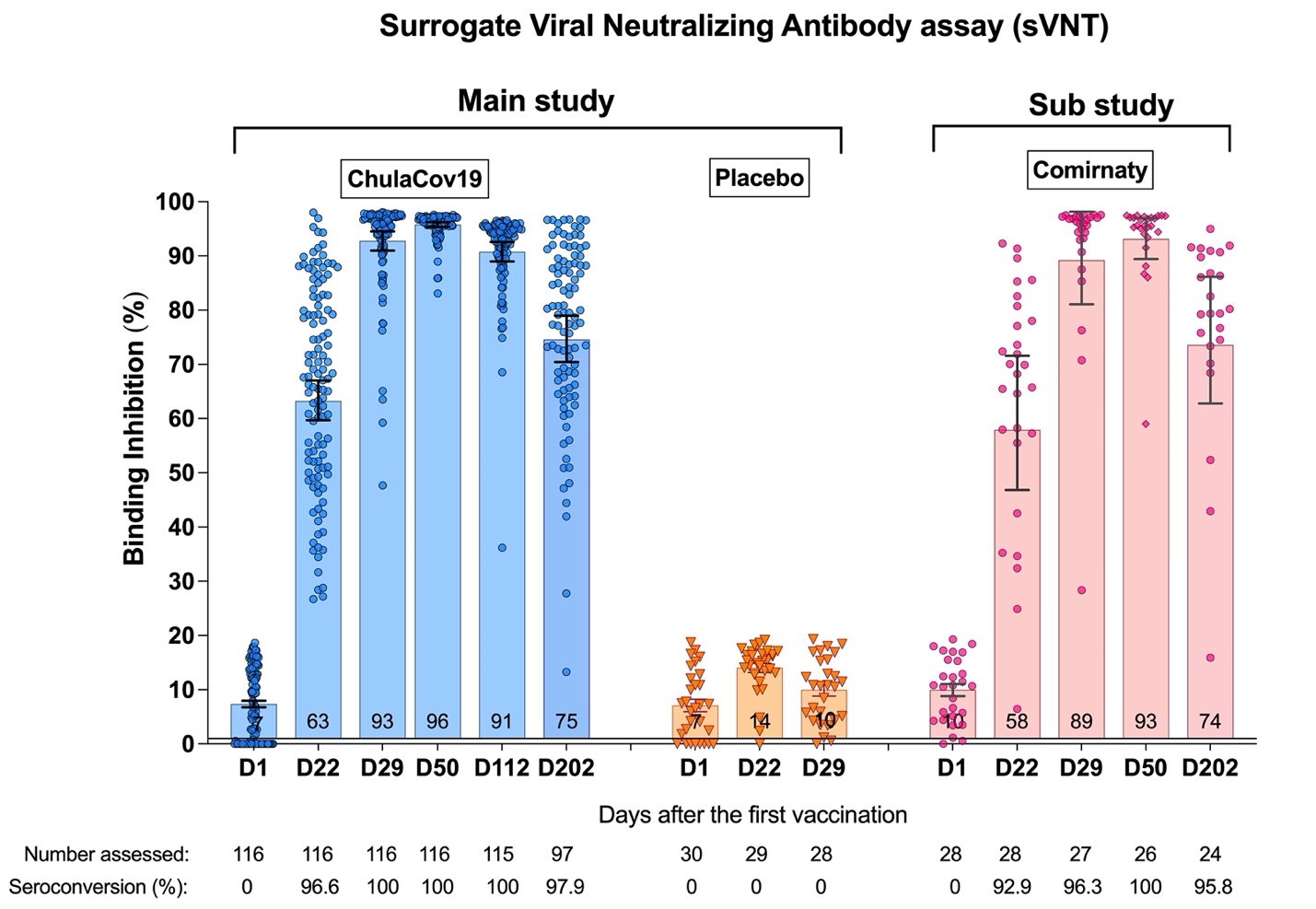

Supplement: Supplementary file 1 — Supplementary Information 1. [file 41598_2023_49653_MOESM1_ESM.docx]
